# Chestnut Lily Beverage (CLB) Processing Using Ultrasound-Assisted Nisin: Microbiota Inactivation and Product Quality

**DOI:** 10.3390/foods11213344

**Published:** 2022-10-25

**Authors:** Yao Cui, Jianxue Liu, Sihai Han, Peiyan Li, Denglin Luo, Jinying Guo

**Affiliations:** 1 College of Food and Bioengineering, Henan University of Science and Technology, Luoyang 471023, China; 2 Henan Food Raw Material Engineering Technology Research Center, Henan University of Science and Technology, Education Department of Henan Province, Luoyang 471023, China

**Keywords:** NUS treatment, chestnut lily beverages, ultrasound treatment, sterilisation, microbial inactivation

## Abstract

We evaluated the effects of ultrasound (US) and ultrasound combined with nisin (NUS) treatments on the properties of chestnut lily beverages (CLB) using conventional thermal pasteurisation (TP) as a control. After CLB samples were treated with US and NUS for 20, 40, or 60 min, the polyphenol oxidase activity (PPO), microbial inactivation effect, colour, pH value, total phenolic content, and antioxidant capacity of the CLB were observed. It was found that the inactivation rate of PPO in CLB after NUS treatment was higher than that in the US, indicating that NUS treatment aggravated PPO inactivation. Treatment time was important in the inactivation of microorganisms by US and NUS; NUS had a lethal synergistic lethal effect on microorganisms in CLB and when compared with US, NUS reduced changes in the CLB colour value. Notably, the total phenolic content and antioxidant capacity of the US- and NUS-treated CLB significantly increased relative to the TP group. These results that suggest NUS has a potential application value in the development of CLB because it reduces the risk of microorganism contamination and helps improve the quality of CLB. This study provides technical support and a theoretical basis for the improved production of CLB.

## 1. Introduction

With an increased attention on human health, the demand for the health characteristics of food is also increasing [1]. In the current market, plant-based beverages have captured the attention of health-conscious consumers [2]. Lily bulb (*Lilium davidii* Duchartre) is an herbaceous plant of the Liliaceae family that has high nutritional and medicinal value. It is rich in biologically active components such as organic acids, polyphenols, and minerals that have anti-fatigue, hypoglycaemic, anti-depression, and antioxidant effects, which are beneficial to human health [3]. Chestnut (*Castanea mollissima*) belongs to the Fagaceae plant and is cultivated in large quantities in China [4]. Chestnut is rich in unsaturated fatty acids that are conducive to preventing cardiovascular diseases [5]. Vitamin C, polyphenols, calcium, magnesium, phosphorus, and potassium in chestnut are all essential nutrients for the human body. It is an attractive strategy to develop new compound beverages from these two raw materials, which can not only draw more attention to the nutritional value of chestnuts and edible lilies, but also reduce product loss during storage and increase their economic value.

Sterilisation is one of the critical steps in beverage processing and minimizing nutrient loss during sterilisation is important for chestnut lily beverages (CLB). The traditional heat sterilisation method can effectively inactivate microorganisms in the juice and improve the shelf life, but the high temperature will cause colour damage and nutrient loss, which will affect the quality of the juice [6]. The field of juice processing, therefore, requires the development of nonthermal food processing technologies that retain as much nutritional properties as possible while maintaining microbial safety [7]. Ultrasound sterilisation (US) is an innovative nonthermal sterilisation method [8], which has shown great potential in controlling the microbial load of fruit juices and reducing endogenous enzymatic activity [9]. The mechanism of microorganismal and enzyme inactivation of the US mainly relies on its mechanical effects (e.g., turbulence, liquid circulation, shock wave, high-speed shear force, and micromechanical shock) caused by cavitation [10], which causes changes in the temperature and pressure of enzymes and microorganisms. This phenomenon causes cell wall and membrane damage, thereby triggering overall cell inactivation [11]. In addition, the US can also produce chemical effects by generating hydrogen radicals through water vapour decomposition. Free radicals can react with enzyme structures or amino acids in microorganisms and affect their activity [12]. In addition, Ordonez-Santos et al. found that the US can effectively retain beneficial nutrients and biologically active compounds even though it has little effect on the physicochemical and sensory indicators of beverages [13].

Using the US alone still has some disadvantages, such as its high energy costs and long processing times, which limit its application in fruit juice processing. However, a combination of ultrasound and other sterilisation techniques has promising potential in fruit juice processing. Ultrasound combined with other sterilisation technology not only exhibited synergistic effects by inactivating microorganisms and reducing enzyme activity, but also helped reduce the high energy cost of the US, but maintained the nutritional, sensory, and physicochemical properties of beverages. Previous studies have investigated the suitability of ultrasound-based sterilisation such as, thermal ultrasound [14], ultrasound plus gas [15], ultrasound plus high pressure [8], ultrasound plus pulsed electric field, and ultrasound plus antibacterial agents, among others [16]. Notably, combined ultrasound sterilisation techniques have also been used in carrot juice [17], milk [18] and infant formula [19] production.

Combined US sterilisation techniques have attracted the attention of researchers due to the simplicity and low cost of ultrasound coupled with antimicrobial agents such as nisin [20]. Nisin is a biological antibacterial safener approved and recommended for food preservation by the World Health Organization. It is in an antibiotic family of antimicrobial peptides produced by *Lactococcus lactis* strains and is widely used in the storage and preservation of liquid foods [21]. To date, there have been no studies on the effects of ultrasound combined with nisin sterilisation technology (NUS) in the production of chestnut lily beverages (CLB).

Sterilizing CLB with nisin in combination with US may save energy costs while maintaining microbiological safety and may have an impact on the physicochemical properties of the CLB. Therefore, this study aimed to evaluate the following effects of NUS on the overall quality of CLB: (i) How thermal pasteurisation (TP), ultrasound (US), and nisin combined with ultrasound (NUS) affects PPO inactivation rates in CLB after treatment. (ii) Do US and NUS have PPO inactivation dynamics? (ii) Comparison of the bactericidal effects of NUS, TP, and US. In addition, the browning index and phenolic compounds are important characteristics of CLB that affect consumer acceptance and beverage quality. Therefore, this study also evaluated the total phenolic content and browning index of CLB after TP, US, and NUS treatments. In this study, by comparing the physicochemical properties of CLB treated with TP, US, and NUS, it provides theoretical and technical guidance for the selection of sterilisation conditions and is helpful for the development of high-quality juices.

## 2. Materials and Methods

### 2.1. Juice Preparation

Lily bulbs were purchased from Jianhuisheng Trading Co., Ltd. (Lanzhou, China) and chestnut kernels were purchased from Da Zhang supermarket (Luoyang, China). The samples were stored at 4 °C until use. The chestnuts and lilies were mixed at a 1:1 (*w*/*w* ratio and blanched at 85 °C for 3 min. This mixture was then combined with distilled water at a ratio of 1:9 (*w*/*v*) before food additives (0.18% citric acid, 0.2% ascorbic acid, 0.06% carboxymethyl cellulose (CMC), 0.08% xanthan gum and 7.5% sugar) were added, and the juice was extracted using a juicer (HR2838Philips, Home Appliances Co., Ltd., Zhuhai, China) for 1 min. The solution was filtered twice through two layers of gauze, followed by homogenisation treatment using a high-shear dispersing homogeniser (AD500S-H, Trillion Instruments Co., Ltd., Shanghai, China) at 12,000 rpm for 6 min. The CLB samples (12.52 °Brix, 4.02 pH, 1.12% protein, 10.1 mg/100 mL Vitamin C) were stored in 500 mL sterile glass bottles at 4 °C until further processing.

### 2.2. Preparation of Bacteriocin Solution

Nisin with a potency ≥900 IU/mg (Shandong Furuida Biotechnology Co., Ltd., Qingdao, China) was dissolved in 100 mL juice and passed through a 0.22-μm filter to remove any microorganisms before being stored at 4 °C for later use.

### 2.3. Sterilisation

The prepared beverage samples were sterilized by TP, US, and NUS, respectively. Specifically, the TP treatment method heated the samples at 85 °C for 10 min using an induction cooker (C22-RT22E01, Midea Group Co., Ltd., Foshan, China). The US treatment followed a method described previously [22]. The juice sample (80 mL) was placed in a 100 mL glass beaker that had been washed three times with sterile water and then with 20 mL of untreated juice to avoid cross-contamination. The application power level of the ultrasonic processor was set to 60% of the maximum power (950 W), with the pulse duration set to 5 s on and 5 s off and used a frequency range of 20–25 kHz. The samples were processed using an ultrasonic processor (JY92-IIdN; Ningbo Scientz Biotechnology Co., Ltd., Ningbo, China). The samples were sonicated for 20, 40, or 60 min. A water bath was used to maintain the temperature at 55 °C, and the temperature of the juice was monitored with a thermometer. For the NUS treatment, nisin was added to the prepared juice at a final concentration of 200 ppm, followed by the described US procedure. Untreated samples were used as the blank control group (CK).

The single treatment volume of juice was 80 mL, and at least three parallel experiments were performed for each treatment. All the tested treatments were performed in the dark to prevent any accidental effects of light on the samples. After sterilisation, all samples were collected, stored in sterilised bottles, and immediately refrigerated at 4 °C until analysis.

### 2.4. Chemical Reagents

The following chemicals were used in this study: polyvinylpyrrolidone (PVPP) and Triton X-100 were purchased from Kema New Materials Co., Ltd. (Ulanqab, China). The 2,2-Diphenyl-1-picrylhydrazyl (DPPH), catechol, quercetin, and gallic acid were purchased from BASF Biotechnology Co., Ltd. (Hefei, China). Folin-Ciocalteu reagent, sodium carbonate, and ascorbic acid were purchased from Luwei Pharmaceutical Co., Ltd. (Qingdao, China). Tric acid monohydrate was sourced from Shandong Ensign Industry Co., Ltd. (Huzhou, China); xanthan gum and carboxymethyl cellulose were purchased from Xinjiang Meihua Co., Ltd. (Wulumuqi, China); sugar was purchased from the Lingyunhai Sugar Industry Group (Rizhao, China); 2,6-dichloroindoxyl, NaCl, phosphate, glacial acetic acid, and sodium hydroxide were purchased from Nanjing Chemical Reagent Co., Ltd. (Nanjing, China). All the culture media were obtained from Nissui Biotechnology Co., Ltd. (Qingdao, China). Distilled water was prepared in the laboratory.

### 2.5. Determination of Enzyme Activity

The enzyme extraction solution was prepared as previously described [23]. Briefly, 4% (*w*/*v*) polyvinylpyrrolidone (PVPP), 1% (*v*/*v*) Triton X-100, and 1 M NaCl were dissolved in 0.2 M phosphate and adjusted to pH = 6.5. The sample and enzyme extraction solution were mixed 1:1 (*v*/*v*) and centrifuged at 14,000 rpm (380R, Hettich Instruments, Germany) at 4 °C for 30 min. The polyphenol oxidase (PPO) activity was determined using a slightly modified method [24]. Then, the prepared supernatant was added to 0.2 mL 2 mM catechol and 2.7 mL 50 mM phosphate buffer (pH 6.8). This mixture was incubated at 37 °C for 5 min to start the enzymatic reaction. A mixture of the blank sample supernatant and the phosphate buffer solution was prepared using the same method. The absorbance at λ = 420 nm was immediately monitored using an ultraviolet-visible spectrophotometer (UV-1800 Shimadzu spectrophotometer) at room temperature (25 ± 1 °C). Enzyme activity was defined as the change in absorbance of each mL of enzyme solution at a 420 nm/min, which was defined as one unit of enzyme activity.

(1) $$\mathrm{Relative}\;\mathrm{activity}=\frac{\mathrm{Activity}\;\mathrm{of}\;\mathrm{treated}\;\mathrm{PPO}}{\mathrm{Activity}\;\mathrm{of}\;\mathrm{untreated}\;\mathrm{PPO}}\times 100\%$$

### 2.6. Mechanical Analysis of Inactivity

Conventional first-order kinetic equations were used, as previously described [25], to predict PPO inactivation in lily juice. An inactivation curve was drawn using t as the abscissa and the logarithm of the relative enzyme activity ln (*t*/*A_0_*) as the ordinate k.

(2) $$\ln\left(\frac{A_{t}}{A_{0}}\right)=-k\cdot t$$

where *A*_0_ is the initial enzyme activity (U/min), *A_t_* is the relative enzyme activity of the sample after t min of treatment (U/min), k is the inactivation rate constant at the studied temperature, min^−1;^ and t is the treatment time (min).

In the half-life of inactivation (*T_1/2_*) equation, *D* is the treatment time required for 90% inactivation of the initial activity under the given conditions, and k is the inactivation rate constant at the studied temperature, min^−1^; and t is the treatment time (min)

(3) $$T_{1/2}=\frac{\ln\left(2\right)}{k}\;$$

(4) $$D=\frac{\ln\left(10\right)}{k}$$

### 2.7. DPPH Radical Scavenging Assay

The antioxidant activity of CLB was estimated using a DPPH radical scavenging assay described previously [26], with slight modifications. DPPH solution (0.12 mmol/L) was prepared by mixing DPPH and methanol in a 100-mL volumetric flask. Fifty microlitres of the sample were mixed with 950 μL 0.12 mmol/L DPPH (methanol solution). The mixture was shaken vigorously and allowed to stand in the dark for 30 min to obtain an extract. The change in absorbance was measured at 517 nm at 25 °C using an ultraviolet-visible spectrophotometer until a stable absorption was achieved. The radical scavenging activity rate was calculated using the following equation:

(5) $$\mathrm{DPPH}\;\mathrm{inhibition}\;\left(\%\right)=\frac{A0-A1}{A0}*100$$

where *A0* is the absorbance of the blank sample, and *A1* is the absorbance of the sample.

### 2.8. Total Phenolic Content

The measurement of total phenolic content (TPC) was adapted from a previously described method [27] to apply the Folin–Ciocalteu method to determine the TPC of the fruit juice samples. A 0.5 mL beverage sample was diluted three times with 10 mL distilled water. Then, 0.25 mL Folin–Ciocalteu reagent and 0.5 mL 7.5% sodium carbonate solution were added to the diluted juice solution. This mixture was incubated in the dark at 20 °C for 30 min. The change in absorbance at 765 nm was measured using a spectrophotometer. Using gallic acid as the standard, the results were expressed as µg gallic acid equivalents (GAE) per 100 mL juice sample.

### 2.9. Ascorbic Acid

We tested the content of ascorbic acid in the samples using the method described previously [28]. A 5 mL sample aliquot was diluted to 100 mL with distilled water at 4 °C before 2 mL diluted juice samples was mixed with 25 mL 20% glacial acetic acid and mixed well by shaking. Then, 2,6-dichloroindoxyl was used to titrate the solution until it was completely pink and did not fade within 15 s. A standard curve was plotted using ascorbic acid standards, and the results were expressed in mg/mL ascorbic acid in the juice samples.

### 2.10. pH, Total Soluble Solids, and Browning Index

The pH of the samples was measured using a pH meter (Mettler-Toledo Instruments Co., Ltd., Shanghai, China). A PAL-1 portable digital refractometer (Atago, Tokyo, Japan) was used to measure the soluble solids content (TSS). The refractometer was calibrated to zero with distilled water before a drop of the beverage was placed on the prism of the refractometer. The TSS content of the beverage was recorded as °Brix. This method was adopted from Meydav et al. [29]. The beverage browning index was determined using the method described above. The juice samples were centrifuged at 4000 rpm for 10 min in a high-speed centrifuge and the supernatant was collected. The absorbance of the supernatant was measured at 420 nm using a spectrophotometer at room temperature.

### 2.11. Microorganisms

The microbial populations in the juice samples were measured as described in the AOAC method [30]. The total bacterial count (TBC) was determined using the nutrient agar inverted plate method. Yeasts and moulds (YM) in the samples were determined by incubating potato dextrose agar medium at 20 °C for 7 days. Purple bile agar plates were incubated at 37 °C for 24 h to count coliforms. The TBC and YM results of the juice samples were expressed as log colony forming units (CFU)/mL, and coliforms were expressed as log (MPN)/mL.

### 2.12. Data Analysis

Data were collected using Statistica (7.0, Statsoft Inc., Tulsa, OK, USA), and images were plotted using Origin (OriginLab Inc., Northampton, MA, USA). Data were analyzed by one-way analysis of variance (ANOVA) followed by Duncan’s multiple comparison using SPSS 16.0 software (IBM Inc., Armonk, NY, USA). The level of significance was set at *p* < 0.05. All experiments were repeated in triplicate

## 3. Results and Discussion

### 3.1. Determination of Enzyme Activity

**Figure 1:**
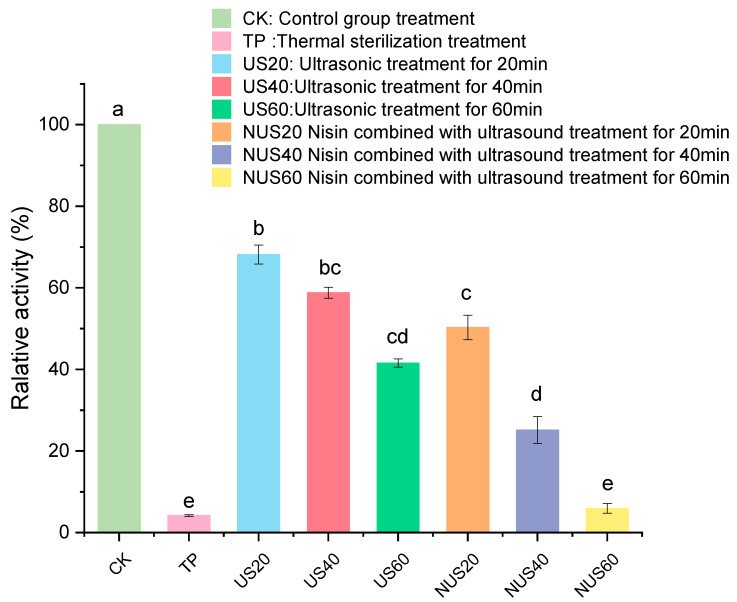
Effects of different sterilisation treatments on the relative PPO activity in CLB. CK represents the untreated group; TP represents the heat treatment group; US20, 40, and 60 represent the ultrasonic treatment groups for 20, 40, and 60 min alone; and NUS20, 40, and 60 represent the ultrasonic combined nisin treatment groups for 20, 40, and 60 min. Different letters indicate significant differences between the means (*p* < 0.05).

Polyphenol oxidase (PPO) is one of the main enzymes involved in the browning of food. PPO catalyses the hydroxylation of monophenols and oxidation of o-diphenols to o-quinones, which subsequently produce undesirable brown pigments [31]. When PPO is mixed with its substrate, the cell structures are destroyed, which can negatively affect the colour, taste, nutritional properties and shelf life of food products [32]. Thus, the inactivation of PPO protects the food product’s colour and prolongs its shelf life [33]. Figure 1 shows that TP treatment had the strongest PPO inactivation effect on CLB when compared with CK, and the PPO activity after TP treatment was 4.2%. The enzymatic activity of PPO decreased as the sonication time increased, indicating that the US had an inhibitory effect on PPO. The PPO activity remained above 40% after treatment with 60 min after ultrasound. Moreover, PPO activity in the nisin combined with ultrasonic treatment for 60 min (NUS60) treatment group was 5.94%. Therefore, the combined treatment with ultrasound and nisin could significantly improve the inactivation of the PPO enzyme. This result may be related to the fact that the assisted sonication process can interfere with the boundary layer on the cell surface and affect diffusion [34].

Ultrasonication disrupts cell membranes and increases the diffusion of antibacterial solutions in the product [35], thereby increasing the effectiveness of ultrasonic waves and altering enzymatic activity. Ultrasound mainly destroys the structure of enzymes via strong shock waves or jets [36]. It was found that when nisin binds to the enzyme, it can form pores in the cell membrane to impact the enzyme structure [37,38]. Chakraborty et al. [39] found that juice composition, enzyme type, pH, and treatment parameters affected enzyme inactivation rates. The quantitative composition of PPO phenolic substrates in different crop varieties can differ, resulting in different inactivation effects [40,41,42,43]. For example, the activity of grape PPOs decreased by about 50% after heat treatment at 65 °C for 20 min, and was completely inactivated after heat treatment at 75 °C for 15 min [44], while mango PPO took more than 15 min at 80 °C to lose 50% of its activity [40]. Additionally, the PPO of lettuce was inactivated after 5 min at 90 °C [45]. Indeed, the temperature during processing is an important factor that significantly affects the catalytic activity of PPO, which may be related to how temperature affects the solubility of oxygen and thus, lead to enzymatic denaturation [44,46]. Therefore, in order to balance the degree of PPO inactivation and its effect on product quality, the treatment conditions of different processes need to be carefully set [47].

### 3.2. Mechanics of Enzyme Inactivation

**Figure 2:**
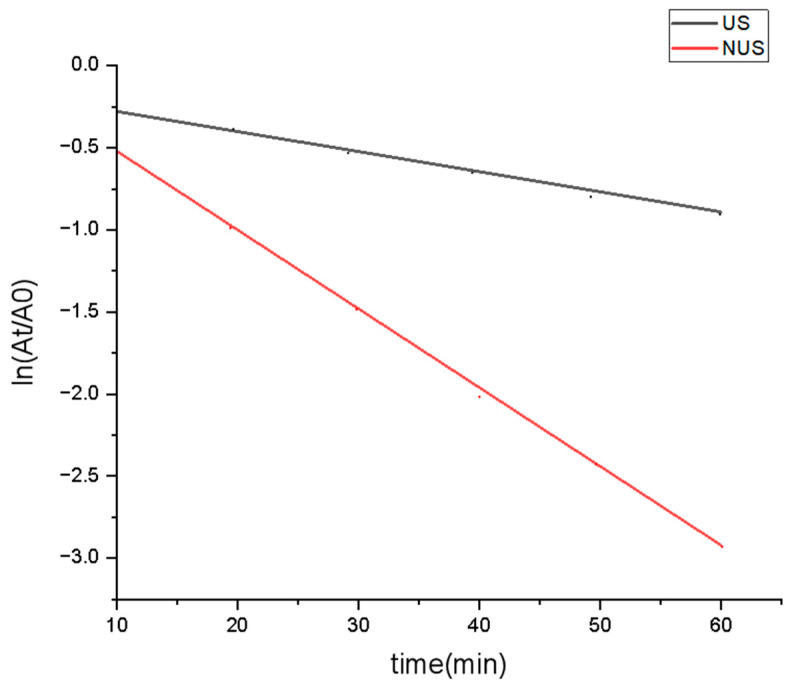
Inactivation curves of US and NUS treated PPO. US stands for ultrasonic treatment, NUS stands for ultrasonic treatment combined nisin treatment, which showed that the degree of PPO denaturation increased with increasing sterilization time.

Thermodynamic parameters of PPO inactivation provide information on the thermostability of the enzyme [48]. The kinetic rate of PPO was illustrated as a logarithmic plot, which showed that the degree of PPO denaturation increased with increasing sterilisation time (Figure 2). This linear relationship showed that the PPO after the US and NUS treatments followed first-order reaction kinetics, with correlation coefficients R^2^ of 0.958 and 0.934, respectively (Table 1). The slope of each curve obtained the kinetic rates were used to calculate the inactivation rate constant *k*, and then the *D* value and half-life_T1/2_ of PPO under different treatments were calculated. The k value of PPO in the US treatment was 1.31 × 10^−2^ min^−1^, and the *k* value reached 4.36 × 10^−2^ min^−1^ in the NUS treatment (Table 1), indicating that the inactivation rate constant of NUS was higher than that of the US treatment. The *D* value of PPO significantly decreased after NUS treatment, and the half-life (*T*_1/2_) of NUS was also significantly reduced compared to that of US. These results indicated that the inhibitory effect of the US alone on PPO was weak, but in combination with nisin, it can achieve a significant inhibitory effect. A previous study reported that physical treatment affects outer membrane permeability [49], which may lead to differences in nisin sensitivity and enzymatic activity. A study by Muñoz et al. [50] showed that the combination of ultrasound with nisin increased the rate of cell destruction, which may affect the inactivation of the enzyme. However, the mechanism of the synergistic enzyme inactivation observed with nisin treatments requires further study.

### 3.3. DPPH Radical Scavenging Assay

**Figure 3:**
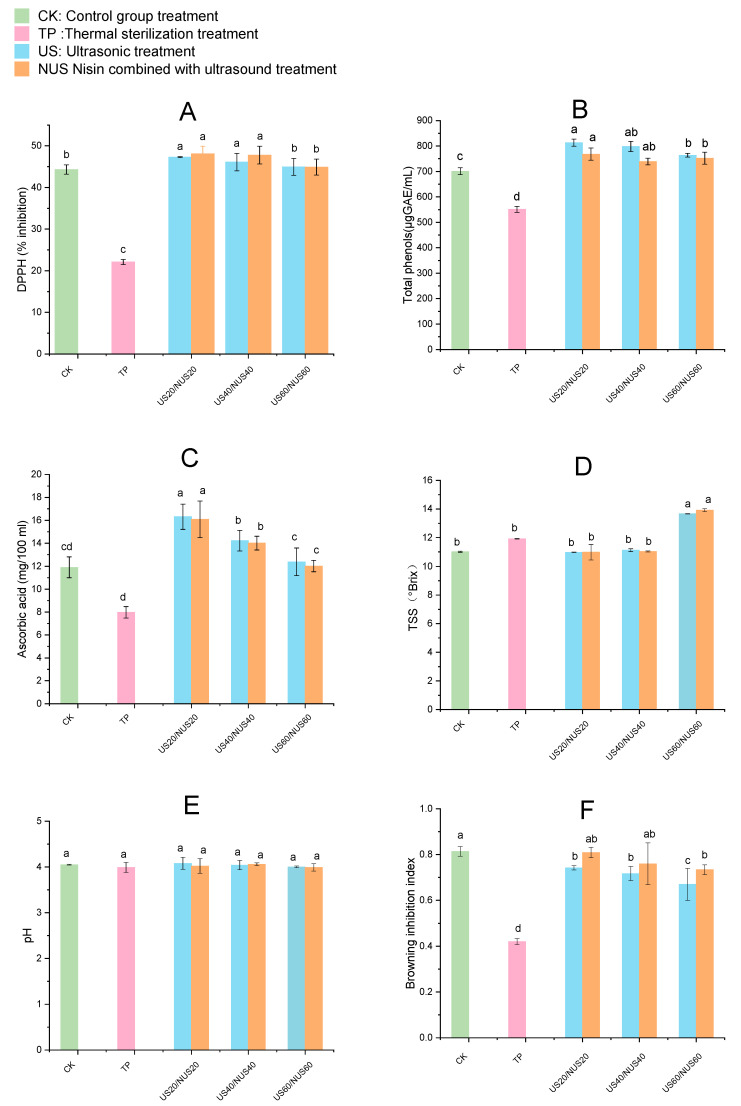
Changes in physicochemical property in chestnut lily beverages treated with different sterilisation techniques. Where (**A**) stands for DPPH inhibition, (**B**) stands for total phenolic content, (**C**) stands for ascorbic acid content, (**D**) stands for pH value, (**E**) stands for TSS content, (**F**) stands for browning Inhibition Index. CK represents the untreated group, TP represents the heat treatment group, US20, 40, 60 represent the ultrasonic treatment groups for 20, 40, 60 min alone; NUS20, 40, 60 represent the ultrasonic combined nisin treatment groups for 20, 40, 60 min. The error bars indicate the standard deviation. Different letters (a, b, c, d) indicate significant differences between the means (*p* < 0.05).

DPPH assay is one of the methods commonly used in the food industry to measure antioxidant capacity. Figure 3A shows the effect of sterilisation on the percent DPPH inhibition in CLB. Compared to the TP group (22.10%), DPPH inhibition was significantly increased in the US20, NUS20, US40, and NUS40 groups. NUS20 had the highest DPPH inhibition (48.11%), followed by US20 (47.31%), of the treatment groups. Except for NUS60 and US60, no significant differences were observed in the other ultrasound treatment groups when compared with CK. In the US and NUS juice samples, the increase in DPPH inhibition could be due to increased extractability of antioxidant compounds such as ascorbic acid, total phenolics and flavonoids [51]. The effects of ultrasound treatment on important nutrients mainly include (i) the destruction of the cell walls and vacuoles in plant tissues or the collapse of colloidal particles due to cavitation and the capillary effect, which both result in the release of soluble nutrients; and (ii) the destruction of the molecular structure of nutrients. In the sterilization process, the former is a much larger contributor than the latter, so the compounds with important nutritional value re-retained to the maximum extent or even increased [52]. Studies of antioxidant activity have been consistent with the effect of ultrasound observed in orange juice [53] and cranberry [54] juices. The decreases antioxidant potential observed in NUS60 and US60 may be related to the increase in hydroxyl radicals that react with phenols during longer treatment periods [55]. Owing to different sample sources and extraction methodologies, antioxidant capacity is a difficult parameter to compare across studies [56]. Additionally, different results were obtained from studies of the antioxidant activity of composite apple juice using a combination of ultrasonic treatment technologies [57]. These studies provide examples of how the steps of different methods and technical combinations in joint treatments can lead to different results.

### 3.4. Changes in the Content of Total Polyphenols Compounds

Phenolic compounds in fruits and vegetables can reduce the risk of diseases caused by oxidative stress [58]. As shown in Figure 3B, total phenolic content (TPC) in the TP-treated juice significantly decreased compared to that in CK, which may have been due to the complex physical and chemical reactions generated at high temperatures, including the release of phenolic compounds, polyphenol degradation, and decomposition and transformation of phenolic substances [59]. Compared with CK, the TPC of the beverage samples after US treatment increased. The total phenolic content increased from 701.05 µg GAE/100 mL to 813.21 µg GAE/100 mL after US20 treatment, and the total phenolic content increased to 768.15 µg GAE/100 mL after NUS20 treatment. This could have been because of the destruction of the plant cell wall during the US process, which released components such as pectin, cellulose, hemicellulose, and lignin, which contributed to the dissolution and release of phenolic compounds into the juice [60]. Another explanation for the increase in phenolic compounds in US treated samples is that the treatment generated nascent hydroxyl radicals that can combine with aromatic rings to produce more phenolic compounds [57]. TPC gradually decreased with prolonged sonication time, which may be related to the degradation of antioxidants in the CLB [61]. A similar phenomenon was observed when using ultrasonics to treat strawberry juice by Jin et al. [62].

### 3.5. Ascorbic Acid

Ascorbic acid can prevent cardiovascular diseases and protect cells from free-radical-induced damage [63]. The effects of sterilisation on the ascorbic acid content of CLB are shown in Figure 3C. The ascorbic acid content in TP samples was significantly reduced than that of the CK, which limited the availability of this ascorbic acid in TP-sterilised CLB. The ascorbic acid content of US20, US40, and US60 samples increased from 11.90 mg/100 mL to 16.31 mg/100 mL, 14.22 mg/100 mL, and 12.38 mg/100 mL, respectively. When the sonication time was the same, there was no significant difference between the ascorbic acid content of the NUS group and the US group. The increase in ascorbic acid in ultrasound-treated CLB samples could be due to the removal of oxygen by cavitation [64]. Furthermore, sonication can prevent the beverage from being affected by high temperatures, which, like oxidation, can cause ascorbic acid degradation. Moreover, a previous study found that the ascorbic acid content of orange juice increased after sonication [53]. In a study of the effect of ultrasound on tomatoes, it was found that the ascorbic acid content was reduced, potentially due to the rupture of the cell wall and organelles during sonication, which released oxygen and caused ascorbic acid oxidation [65]. These differing results may be related to the ultrasound conditions and the physical and chemical properties of the fruit itself [66].

### 3.6. TSS and pH

The pH and TSS of the samples after sterilisation are presented in Figure 3D,E. After all treatments, the pH of the beverage did not change significantly compared to that of the control group (*p* < 0.05). In general, the pH of CLB was relatively stable after the different sterilisation treatments. Although the TSS content significantly increased after US60 and NUS60 treatments (*p* < 0.05), there was no significant difference in the TSS content of the US20, US40, NUS20, and NUS40 treatments groups when compared with the CK. The increase in TSS content may be related to the rupture of the cell wall and the release of phenolic substances in the vacuoles after long-term ultrasound treatment, resulting in the release of intracellular compounds [67], while short-term US treatment did not significantly affect these physicochemical properties. Similar changes were observed in the sonication of tomato [65] and orange juice [68].

### 3.7. Browning Inhibition Index

The browning is an important parameter that reflects the quality of food, as a pleasant colour plays an important role in consumer appreciation. The effects of sterilisation on the browning inhibition index of CLB are summarised in Figure 3F. Compared with the control group, the browning inhibition index of all juice samples after sterilisation treatment was significantly decreased (*p* < 0.05), and the sterilisation treatment increased the degree of browning of the beverage. The increased browning was most significant after TP treatment, perhaps because high temperatures can lead to an excessive loss of antioxidants in CLB [62]. The browning inhibition index decreased with increasing ultrasound treatment time, indicating that the ultrasound processing time could influence juice colour. The degree of browning in the NUS group was lower than in the US treatment group, which could be because the combined action of sterilant and ultrasound enhanced the inactivation of enzymes related to enzymatic browning, thereby significantly reducing enzymatic browning in fruit juice [69]. These results are consistent with a previous study that investigated the browning index of sonicated orange juice [36]. PPO activity was found to be significantly correlated with food colour and browning [70]. In addition, it has been found that the hydroxyl radicals generated during the ultrasonication process can lead to the hydroxylation of the ortho, median, and para positions of the aromatic rings in phenolic compounds, which may also affect the visible spectral area of the fruit juice, causing a change in the browning inhibition index [71].

### 3.8. Microorganisms

**Figure 4:**
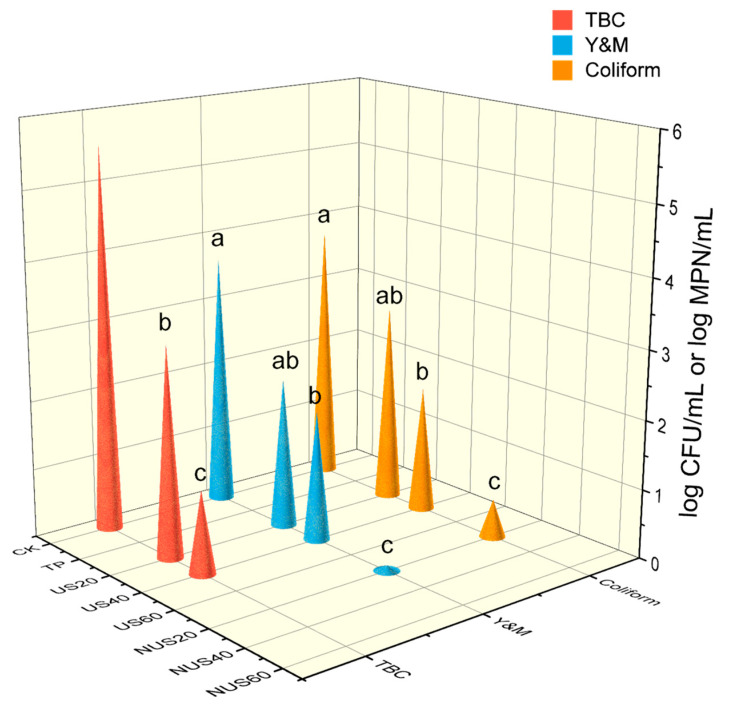
The effects of different sterilisation treatments on microorganism load in chestnut lily beverages. CK represents the untreated group; TP represents the heat treatment group; US20, 40, and 60 represent the ultrasonic treatment alone groups for 20, 40, and 60 min; and NUS20, 40, and 60 represent the ultrasonic combined nisin treatment groups for 20, 40, and 60 min. The *Escherichia coli* results are expressed as log MPN/mL, while the other results were expressed as log CFU/mL. Values with different letters (a, b, and c) are significantly different from one another (*p* < 0.05).

The effects of sterilisation treatment on CLB microorganisms are shown in Figure 4. All sterilisation treatments reduced the amount of TBC, Y&M, and coliforms, but complete inactivation of microorganisms was observed only in TP, US60, NUS40, and NUS60 treatment groups. TP is still a rapid and effective sterilisation method because high temperatures destroy bacterial proteins, nucleic acids, and enzyme systems, resulting in microbial inactivation [72]. Under the same treatment time, the inactivation of microorganisms was higher in the NUS treatment group than that of the US group, which indicates that nisin improves the biological inactivation effect of ultrasound. This could be related to sonication and the accumulation of nisin’s physical and chemical effects that accelerated the damage of microbial cells [52]. The same conclusion was found by Liao et al. in a study of nisin and thermosonication treatment of fresh apple juice, in which the decrease in the number of microorganisms in the sample after US treatment was related to cavitation [52]. The sterilisation mechanism of US is related to transient and stable cavitation [73]. Stable cavitation can lead to microflow, which can generate stress on microorganisms. Instantaneous cavitation causes the irregular oscillation of bubble formation, which increases the local temperature and pressure in the product, resulting in the implosion of bubbles and the generation of shear force and microjets in the liquid, which causes cell membrane disintegration and enzyme inactivation [74]. Cavitation can also cause chemical changes that split water molecules to form H+ and hydroxide radicals. The recombination of H+ and hydroxyl radicals form hydrogen peroxide, which has bactericidal properties [75]. In this study, we observed that the effect of microbial sterilisation was correlated with sonication time, which was potentially related to the energy of the ultrasound being absorbed and converted into heat during long treatment times [76]. The mechanism of nisin sterilisation causes ion leakage and ATP hydrolysis, which leads to cell death [38]. Nisin can also interfere with the biosynthesis of the cell wall, which prevents the synthesis of peptidoglycan, the main component of the cell membrane, from lipid II, leading to cell destruction and death [77]. The antibacterial effect of nisin is related to its concentration, pH, and temperature [77]. In fact, some previous studies have reported the synergistic effects of inactivating microorganisms using combined technology approaches [78], similar to those observed in the number of TBC, yeasts, and moulds of apple juice treated with nisin combined with sonication [79]. Ultrasound combined with high-pressure treatment has been reported to be more effective than sterilisation alone [80]. Similarly, the inactivation rate of microbial cells was higher in apple juice treated with UV irradiation in combination with pulsed light [81]. The same phenomenon was also observed in carrot juice treated with high-pressure carbon dioxide and hydrostatic pressure [82]. In conclusion, combining ultrasonic and sterilisation technologies has potential application in reducing microorganismal contamination in processed beverages.

## 4. Conclusions

This study comprehensively explored the quality attributes of CLB subjected to different bactericidal treatments, including microbial measurement, enzyme activity, physicochemical indicators, functional indicators, and antioxidant capacity. Compared with US treatment, NUS treatment shortened and improved PPO inactivation time and efficiency. Under conditions that must ensure the microbial safety of CLB, NUS can not only maintain the sensory quality and physicochemical parameters of fruit juice products, but also improve their functional characteristics. The results of this study indicated that NUS technology is a new potential nonthermal sterilisation technology. This information is valuable for the food industry because it further develops sterilisation methods using ultrasound, which can help reduce energy consumption during processing and improve the inactivation of microorganisms in food. It can also help the food industry to produce higher-quality juices and has promising commercial applications. It should be noted that this paper mainly studied the immediate observational effects of sterilisationm, therefore, its storage conditions, extended shelf life, and various conditions for industrial application should be further examined in the future.

## Figures and Tables

**Table 1:** Values of *k*, *D*, and *T*_1/2_ of PPO inactivation in chestnut lily beverages treated with US and NUS.

	*K*/(10^−2^ min^−1^)	*D*/min	*T*/_1/2_ min	*R* ^2^
**US**	1.31 × 10^−2^ min^−1^	175.5 ± 7.30	52.8 ± 2.21	0.958
**NUS**	4.36 × 10^−2^ min^−1^	52.6 ± 1.51	15.9 ± 0.45	0.934

Note: *T*_1/2_: treatment time required for 50% inactivation of the initial activity under given conditions; *D*: treatment time required for 90% inactivation of initial activity under given conditions; *K*: inactivation rate constant.

## Data Availability

The datasets generated for this study are available on request to the corresponding author.
